# Effect of Temperature and Load on Tribological Behavior in Laser-Cladded FeCrSiNiCoC Coatings

**DOI:** 10.3390/ma16083263

**Published:** 2023-04-21

**Authors:** Haiyang Long, Wei Hao, Rucheng Ma, Yongliang Gui, Chunyan Song, Tieyu Qin, Xuefeng Zhang

**Affiliations:** 1 School of Metallurgy and Energy, North China University of Science and Technology, Tangshan 063210, China; 2 Tangshan Key Laboratory of Special Metallurgy and Material Preparation, North China University of Science and Technology, Tangshan 063210, China; 3 Institute of New Materials, Guangdong Academy of Sciences, National Engineering Laboratory for Modern Materials Surface Engineering Technology, Guangdong Provincial Key Laboratory of Modern Surface Engineering Technology, Guangzhou 510650, China; 4 Tanggang Technology Center of Hegang, Tangshan 063611, China

**Keywords:** FeCrSiNiCoC, laser-based cladding, coating, wear mechanism

## Abstract

The FeCrSiNiCoC coatings with fine macroscopic morphology and uniform microstructure were made on 1Cr11Ni heat resistant steel substrate by a laser-based cladding technique. The coating consists of dendritic γ-Fe and eutectic Fe-Cr intermetallic with an average microhardness of 467 HV_0.5_ ± 22.6 HV_0.5_. At the load of 200 N, the average friction coefficient of the coating dropped as temperature increased, while the wear rate decreased and then increased. The wear mechanism of the coating changed from abrasive wear, adhesive wear and oxidative wear to oxidative wear and three-body wear. Apart from an elevation in wear rate with increasing load, the mean friction coefficient of the coating hardly changed at 500 °C. Due to the coating’s transition from adhesive wear and oxidative wear to three-body wear and abrasive wear, the underlying wear mechanism also shifted.

## 1. Introduction

As one of the three failure modes of materials, wear causes huge economic losses to the national economy and society [1,2]. Consequently, the development of low-cost and high-performance wear-resistant materials has become a focus of attention [3,4].

Fe and Si are abundant, inexpensive resources, so new classes of heat-resistant ferritic steels are being developed, and the Fe-Cr-Si ternary system is among the promising candidates [5]. The addition of Cr to steel can strongly improve its heat-resistance and corrosion-resistance [6,7,8,9,10,11,12], and the addition of Si to Fe and Fe-Cr steels results in the enhancement of a SiO_2_ protective layer on the surface, thus greatly enhancing the high-temperature oxidation resistance [13,14]. Fe-Cr-Si alloy material has metal silicides represented by Fe3Si, which has characteristics such as high hardness, strong interatomic bonding and good wear resistance [15,16,17]. Yuan and Wang [18] prepared an α/Fe_9_Cr_9_Si_2_ ternary metal silicide alloy, which had a low coefficient of friction under dry sliding friction conditions. Three kinds of Fe-Cr-2Si alloys were designed by Leong et al. [19], who experimented with their oxidation resistance in high-temperature steam. The Fe20Cr2Si alloy exhibited the best oxidation resistance of the three in high-temperature steam. Moon et al. [20] generated Fe-Cr-Si alloy specimens and tested them for oxidation at 1200 °C. The samples displayed superior oxidation resistance on account of the dense Cr_2_O_3_ and SiO_2_ layers. From these studies, it is clear that Fe-Cr-Si system materials perform excellently under the conditions of wear and high-temperature oxidation. Unfortunately, the brittleness of Fe-Cr-Si alloys seriously limits their development. To overcome this defect, the addition of further alloying elements to Fe-Cr-Si alloys to improve their properties combined with the use of laser cladding technology to prepare the coating is a good method. The addition of Ni and Co to the alloy promotes structural transformation from Bcc to Fcc and reduces the brittleness of Fe-Cr-Si alloys [21]. The addition of C to the alloy promotes the formation of carbides and enhances its wear resistance [22].

Laser-based cladding technology, which is one kind of laser-based deposition technique, has been intensively studied for decades [23,24]. Cladding powder undergoes fast melting and solidification on the substrate surface under the intense laser beam, resulting in a covering [25,26]. With a minimal heat-affected zone, the coating may successfully metallurgically attach to the substrate [27,28,29]. Furthermore, laser-based cladding technology has advantages such as fine microstructure, low dilution rate, environmental friendliness and material saving [30,31,32]. Researchers have discovered that the stress self-adaptation property of Fe/Mn/Si/Cr/Ni coatings can significantly enhance their wear resistance [33]. Therefore, laser-based cladding technology is a promising method for fabrication of FeCrSiNiCoC coatings with extraordinary performance on the surface of mechanical parts [34,35].

In conclusion, the Fe-Cr-Si system using laser cladding has excellent properties in high temperature oxidation resistance and wear resistance, but the wear resistance under high temperature and serious load is less studied. Accordingly, in the present study, this laser-based cladding method was further explored, resulting in a brand new coating of FeCrSiNiCoC being applied to the surface of a 1Cr11Ni heat resistant steel substrate. The microstructure and tribological performance of coatings were systematically and thoroughly investigated at different temperatures and loads.

## 2. Experiments

### 2.1. Material of Raw Ingredients and Manufacturing Process

**Figure 1:**
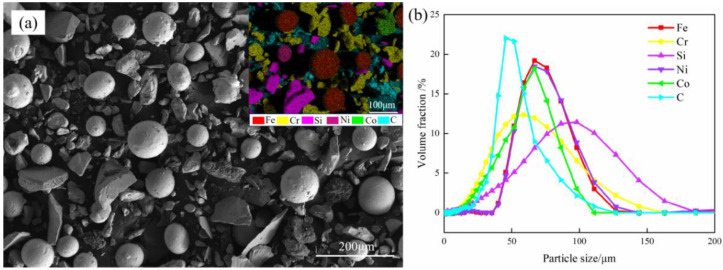
FeCrSiNiCoC mixed powder: (**a**) SEM morphology and EDS; (**b**) particle size distribution.

During this study, as the substrate, 1Cr11Ni2W2MoV(1Cr11Ni) heat-resistant steel was used, with a size of 100 mm × 100 mm × 15 mm. The nominal chemical composition of the FeCrSiNiCoC powder and 1Cr11Ni heat resistant steel substrate are shown in Table 1. For the purpose of enhancing the performance of the laser cladding, the substrate surface was burnished with an angle grinder and cleaned with acetone and alcohol. Figure 1a shows the mixed metal powders used for coating containing Fe, Cr, Si, Ni, C, Co with high purity. The morphology of FeCrSiNiCoC mixed powder is described in Figure 1b. The FeCrSiNiCoC mixed powder particle size distribution was measured by Mastersizer-3000-type diffraction particle analyzer. In order to ensure the feeding effect and reduce the influence of fluidity of powder on the laser cladding forming performance, the selected powder particle size should not be less than 50 μm. The specific particle sizes of various powders are shown in Table 2.

To create the FeCrSiNiCoC coatings, a laser metal deposition system from TRUMPF GmbH in Ditzingen, Germany was used, coupled with a TruDisk 6006 fiber laser. This equipment has a maximum output of 6 kW and a peak beam diameter of 5.39 mm. High-purity helium and argon were selected as the powder feed gas and powder protection gas, respectively, during the laser cladding procedure, because of their high purity. After a systematic study of laser cladding parameters, 1800 W laser power, 4 mm laser spot diameter, 480 mm/min laser scanning speed, 17 mm defocusing, 11.5 g/min (7 r/min) powder conveying speed and 50% overlap rate were determined as manufacturing parameters.

### 2.2. Materials Characterization

The 10 mm × 10 mm × 3 mm specimens were removed from the cross-section of the laser cladded by the wire electrical discharge machining (WEDM) method. All specimens were ground one by one with SiC sandpaper of different grit sizes from 400 to 2000 and polished with diamond polish to achieve a surface roughness of 0.15 µm or less. Then the polished specimens were etched with HF:HNO_3_:H_2_O = 1:6:7 solution for 10 s to 15 s. An optical microscope (OM, Leica DmirmMW-550, Leica Microsystems, Wetzlar, Hessian, Germany) was used to observe the surface profile at low magnification and a scanning electron microscope (SEM, Nova NanoSEM430, FEI, Hillsboro, OR, USA) was employed to characterize the microstructure of the specimen at high magnification. Furthermore, the elements distribution of the laser-cladding coating was analyzed using energy dispersive spectroscopy (EDS, Nova NanoSEM430, FEI, Hillsboro, OR, USA). The FeCrSiNiCoC coatings were heated to 50 °C, 300 °C, 500 °C, 700 °C and held for one hour. The phase composition of the FeCrSiNiCoC coatings at different heat treatment temperatures was analyzed using X-ray diffraction (XRD, Smartlab-9KW, Rigaku Corporation, Tokyo, Japan) with scans ranging from 20° to 110° and settings of 40 kV and 30 mA.

### 2.3. Mechanical Properties

The longitudinal microhardness of a laser-cladded specimen from FeCrSiNiCoC coating to 1Cr11Ni substrate was measured using a Durascan-70G5 Vickers microhardness tester with a load of 500 g and a loading time of 15 s.

Wear property experiments of FeCrSiNiCoC coating were conducted on a MRH-1 slide mode wear tester, in which the laser-cladded sample was pressed under applied load against the outer periphery of a friction ring. After the machine was warmed up to the set temperature, the load was applied to start the wear test. The friction ring was a hollow ring with an outer diameter of 40 mm and an inner diameter of 16 mm made of 2Cr13 stainless steel. Rectangular bodies with the size of 20 mm × 10 mm × 10 mm were removed from the laser-cladded material as the wear test specimens. In preparation for the wear test, each sample was finely ground and polished. The wear-test conditions at different temperatures were as follows: 60 min wear time, 0.209 m/s sliding speed, 753.6 m total wear length, 200 N load, temperatures of 50 °C, 300 °C, 500 °C and 700 °C. The wear-test conditions under different loads were as follows: 60 min wear time, 100 r/min speed, 753.6 m total wear length, 500 °C temperature, loads of 100 N, 200 N, 300 N and 400 N. The weight loss of the specimen after each experiment was measured by a balance with a precision of 0.1 mg. During the wear test, the machine computed autonomously the value of the coefficient of friction (COF). The wear rate (g/m) was calculated using the mass loss (g) and total sliding distance (m) [36]. Finally, SEM and EDS analyses of the worn surface’s shape and component dispersion provide light on the wear process.

## 3. Results and Discussion

### 3.1. Microstructure of Laser-Cladded FeCrSiNiCoC Coating

**Figure 2:**
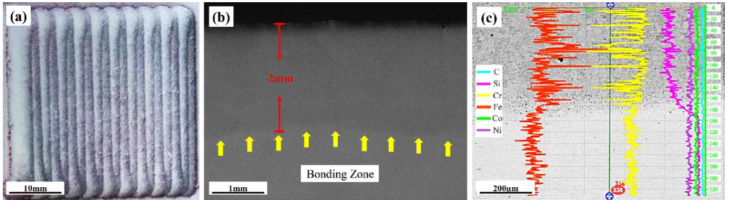
Laser cladded sample (**a**), FeCrSiNiCoC coating and substrate cross section morphology (**b**), and longitudinal element distribution (**c**).

The cross-sectional shape and elemental distribution of a covering made of FeCrSiNiCoC are shown in Figure 2. Figure 2a reveals that the surface of the FeCrSiNiCoC coating is good with no visible cracks. Figure 2b displays the cross section of laser-cladded samples: from top to bottom it shows the FeCrSiNiCoC coating, heat-affected zone andsubstrate. Combined with the hardness distribution, it can be seen that the coating and heat-affected zone thicknesses are around 1.66 mm and 0.34 mm, respectively. Due to the slagging effect of Si element, when the coating melts, it moves freely and wets the steel substrate completely [37]. Therefore, the FeCrSiNiCoC coating is fully dense and homogenous without pores. The EDS line scanning shown in Figure 2c depicts the stable diffusion of alloying elements from the FeCrSiNiCoC coating to the 1Cr11Ni heat resistant steel substrate. It can be observed that the quality of bonding between the coating and the substrate is good.

**Figure 3:**
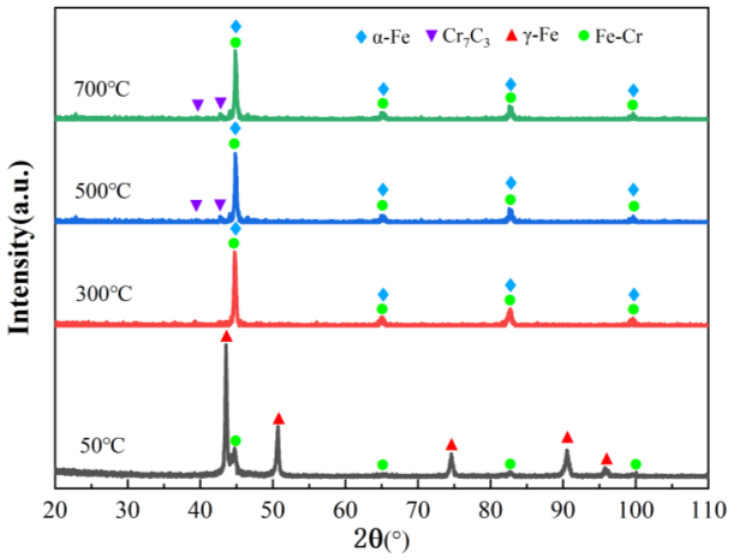
XRD spectrum of the FeCrSiNiCoC coating at different temperatures.

**Figure 4:**
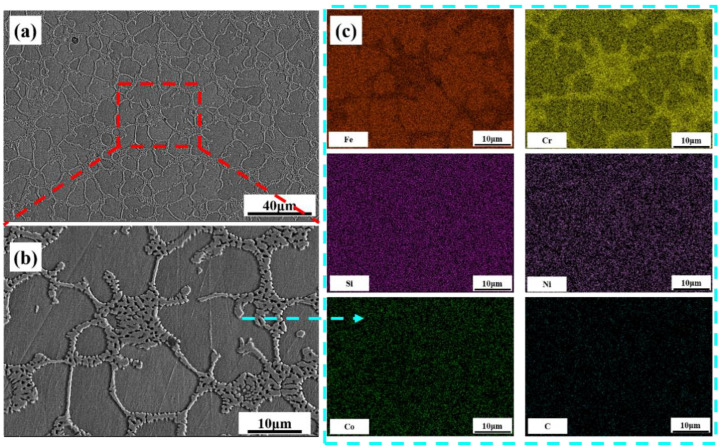
Microstructure of the FeCrSiNiCoC coatings at 50 °C: (**a**) low magnification and (**b**) high magnification SEM image; (**c**) EDS mapping.

Figure 3 presents the XRD profiles of the FeCrSiNiCoC coating at different temperatures. XRD findings indicate that, the FeCrSiNiCoC coating at 50 °C consists mostly of γ-Fe phase and Fe-Cr phase. SEM images and main elements distribution of the FeCrSiNiCoC coatings at 50 °C are shown in Figure 4. Dendritic and eutectic structures can be found in Figure 4, showing that the coating is made up of these types of structure. The EDS results show that coating components are evenly distributed with respect to Fe and Cr. The Fe and Cr contents in the dendrites are 66.47% and 16.04%, respectively. In the eutectic structures, the Fe and Cr contents are 36.02% and 41.09%, respectively. In conjunction with the XRD findings, the conclusion can be drawn that the dendritic structures contain γ-Fe and the eutectic structures contain intermetallic Fe-Cr. When the temperature was 300 °C, the phase of the FeCrSiNiCoC coating changed from γ-Fe, Fe-Cr to α-Fe, Fe-Cr. When the temperature was raised to 500 °C and 700 °C, carbides Cr_7_C_3_ precipitated from the grain boundary.

### 3.2. Microhardness

**Figure 5:**
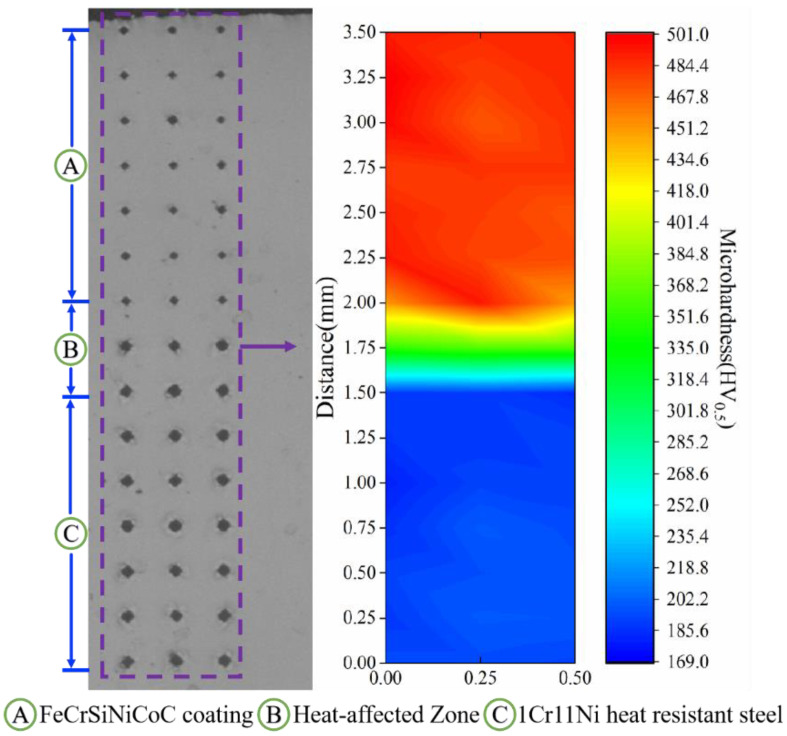
Microhardness distribution diagram from the top of the FeCrSiNiCoC coatings to the 1Cr11Ni heat resistant steel substrate.

Figure 5 depicts the spread of microhardness from the coated surface to the substrate. The mean microhardness values of the FeCrSiNiCoC coating, heat-affected zone and 1Cr11Ni heat resistant steel substrate are 467 HV_0.5_ ± 22.6 HV_0.5_, 335 HV_0.5_ ± 16.6 HV_0.5_, 185 HV_0.5_ ± 5 HV_0.5_, respectively. It is worth noting that there are extremely obvious fluctuations in microhardness between the coating and the substrate (region B in Figure 5). On the one hand, this resulted from the fact that it is the Si component of the alloy powder that diffuses into the substrate and provides the solid solution enhancing effect. On the other hand, in the laser-cladding process, the coating had a large temperature gradient during cooling and solidification [38], which was equivalent to quenching treatment.

### 3.3. Effects of Temperature and Load on Tribological Properties of Coatings

**Figure 6:**
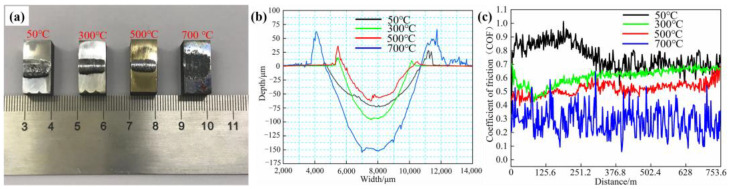
Different temperatures under 200 N: (**a**) surface of FeCrSiNiCoC samples after wear test; (**b**) measurements of typical wear surface morphology using sample wear track sections; (**c**) COF distance curves of samples.

Figure 6a shows the wear-test samples of the FeCrSiNiCoC coating at different temperatures (50 °C, 300 °C, 500 °C and 700 °C) under 200 N load. Figure 6b reveals the surface morphology from the wear-test samples. Wear width, wear depth and wear rate of coatings at different temperatures are shown in Table 3. As the temperature increases, the untreated surface of the coating changes from silver-white, then yellow to dark black No obvious oxide film is visible in Figure 6a, which proves that the FeCrSiNiCoC coating has good high temperature oxidation resistance. As exhibited in Figure 6b, the wear depth and wear width of the coating at 700 °C is greater than for the other test samples, and it can be seen that the coating is not suitable for working at too high a temperature. At 500 °C, the grain size of the coating is reduced and carbide Cr_7_C_3_ precipitated at the grain boundaries, resulting in an increase in the hardness of the coating and a significant improvement in wear performance (See Figure 3 and Supplemental Material for a discussion of the effect of temperature on the organization and properties of FeCrSiNiCoC coatings). Therefore, the coating at 500 °C exhibited the lowest wear breadth, the smallest wear depth, and the smallest wear rate.

Figure 6c depicts the temperature dependence of the coefficient of friction (COF) distance curves for 200N (50 °C, 300 °C, 500 °C and 700 °C). The wear-test samples at 50 °C, 300 °C and 500 °C reached stable wear stages around 240 m, 100 m and 100 m, respectively. However, the friction coefficient of the wear-test sample at 700 °C basically remains within a stable range, as the initial stage of wear is stable wear. The samples maintained a dynamic equilibrium during the wear process. Therefore, under the same load, the friction coefficients of the wear-test samples at 50 °C, 300 °C, 500 °C and 700 °C can be calculated as 0.76, 0.62, 0.50 and 0.29, respectively. When the temperature rises, the friction coefficient drops and the distance to the steady wear stage shortens. However, variations in temperature amplified fluctuations in the friction coefficient, which was caused by the wear surface softening and suffering serious plastic deformation under high temperature sliding friction; the worn surface was seriously bonded and a large amount of spalling occurred, thus resulting in the worn surface becoming uneven.

**Figure 7:**
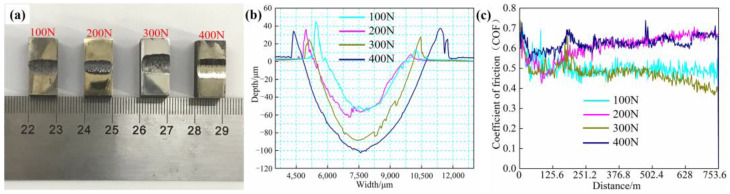
Different loads under 500 °C: (**a**) surface of FeCrSiNiCoC samples after wear test; (**b**) measurements of typical wear surface morphology using sample wear-track sections; and (**c**) COF distance curves of samples.

Figure 7a depicts the wear-test samples of the FeCrSiNiCoC coating at various loads (100 N, 200 N, 300 N, 400 N) under 500 °C temperature. Figure 7b reveals the surface morphology from the wear test samples. Table 4 displays the average wear breadth, wear depth, and wear rate of coatings over a range of temperatures. The wear width and wear depth of coating gradually increase with the increase of load, as illustrated in Figure 7a. As can be observed in Figure 7b, wear does not proportionally increase with a growing load, but shows a slowing trend.

The curves of the coefficient of friction (COF) distance at 500 °C and various weights are shown in Figure 7c (100 N, 200 N, 300 N, 400 N). The wear coefficient fluctuates relatively little for the 100 N and 200 N wear test samples after stable wear. Due to the increased load, the presence of plenty of wear debris caused the frictional wear curve of the 300 N and 400 N wear-test samples to change in the direction of increase after steady wear. All wear test samples enter the stable wear period at around 200 m. Thus, at the same temperature, the average friction coefficients of the wear-test specimens at 100 N, 200 N, 300 N and 400 N in stable wear can be calculated as 0.50, 0.60, 0.47 and 0.66, respectively. The coefficient of friction of the 400 N wear-test sample increased slightly and there were large fluctuations at the stable wear stage, which may be due to the serious plastic deformation of the coating induced by the high load and a large amount of debris.

According to the above results, the FeCrSiNiCoC coating has the best comprehensive wear performance at 200 N and 500 °C, with a wear rate of 2.3 × 10^−5^ g/m. At this time, compared with the Fe_50_Cr_40_Si_10_ coating, the wear rate of the FeCrSiNiCoC coating under higher load and higher temperature is about twice as low as that of the Fe_50_Cr_40_Si_10_ coating [39].

### 3.4. Wear Mechanism

To explore the wear mechanism of the FeCrSiNiCoC coating at various temperatures and loads, SEM and energy spectrum analysis were used to study the worn surface.

**Figure 8:**
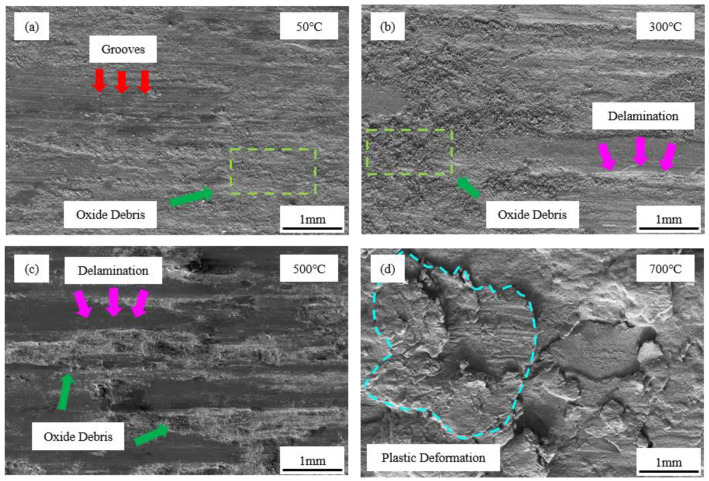
Worn surface morphologies of FeCrSiNiCoC coating at (**a**) 50 °C, (**b**) 300 °C, (**c**) 500 °C, and (**d**) 700 °C.

In Figure 8, we see SEM pictures of the surface wear of 200 N-loaded wear-test specimens at 50 °C, 300 °C, 500 °C, and 700 °C. Figure 8a shows that the worn surface is relatively flat, with more tiny grooves parallel to the sliding direction (pointed by red arrows). This results from the serious shearing of the coating surface under pressure, which causes it to be separated by the micro-cutting action. The soft metal phase in the coating is stripped by friction, thus making the surface uneven. This leads to an increase in contact pressure on the worn surface and a rise in temperature up to the melting point. Some of the debris may be pulled out by kinetic forces and others embedded in the grooves. As a result, the primary wear mechanisms in this instance were abrasive wear and adhesive wear.

Compared with Figure 8a, the coating in Figure 8b shows a little delamination (pointed to by pink arrows) and a substantial quantity of oxidized debris on the surface. The temperature of the contact surface of the friction pair rises as wear progresses and the coating surface partially softens or melts. The softened surface is plastically deformed by the pressure of the friction ring resulting in delamination. Meanwhile, some soft metal phases are stripped from the coating and gradually form oxidation debris as a result of extrusion and oxidation during wear. During the wear process of relative sliding, the surface cutting effect is reduced by the softened wear surface and a large amount of oxidized debris, resulting in a reduction in the friction coefficient and wear rate. As such, oxidation wear and adhesive wear were predominant at 300 °C.

As observed in Figure 8c, the delamination of the surface is even more serious with higher temperatures, with massive accumulation of oxide wear debris on the part of coating near the wear track. However, a large quantity of oxidation debris is produced to replace the worn coating, and smoother wear markings serve to shield the coating from further damage, hence decreasing the friction coefficient and wear rate. In this case, the two most common types of wear are oxidation and adhesive.

Serious plastic deformation and delamination of coating surface can be observed in Figure 8d (represented by the blue region). Under high temperature sliding friction, the worn surface melts and suffers from serious plastic deformation, resulting in serious bonding of the wear surface and massive spalling of hard metal silicide. This makes the wear rate much higher and the friction coefficient fluctuates significantly, but the friction coefficient is greatly reduced. The oxygen content of the worn surface has been reduced due to the peeling off of the hard phase (Table 5). Consequently, the principal wear processes include three-body wear and oxidation wear.

The examination of worn surface shape and wear processes at various temperatures indicates that high-temperature friction leads to the softening of the coating surface and the production of extensive quantities of oxides; this significantly enhances the high-temperature wear resistance of the FeCrSiNiCoC coating. The wear mechanism of the coating shifts from abrasive wear, adhesive wear and oxidation wear to oxidation wear and three-body wear.

**Figure 9:**
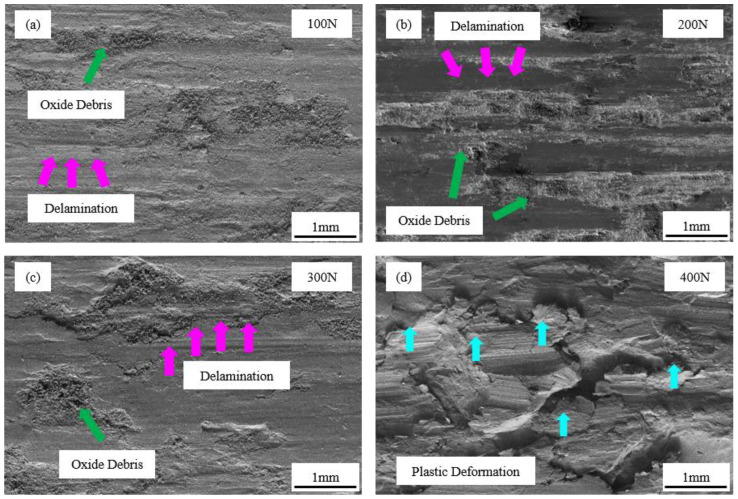
Worn surface morphologies of FeCrSiNiCoC coating at (**a**) 100 N, (**b**) 200 N, (**c**) 300 N, and (**d**) 400 N.

Figure 9 reveals worn surface images captured by scanning electron microscopy from wear-test specimens subjected to 100 N, 200 N, 300 N and 400 N at a temperature of 500 °C. From Figure 9a–c, it is clear that the delamination of the wear surface is gradually becoming more serious and the surface oxidation debris is gradually increasing. As the positive pressure increases, the plastic deformation and flaking of the coating becomes more intense and produces more oxide debris. A large amount of oxidation debris separates coating from the friction ring and acts as a lubricant instead of wear, which causes the wear rate of the coating to gradually increase, but the coefficient of friction remains steady. Oxidation and adhesion wear were the coating’s wear processes.

Figure 9d shows that serious plastic deformation and fine grooves occur on the wear surface at 400 N. Under the combined effect of high temperatures and high loads, the interface between the coating and the friction ring generates high temperatures; this results in a possible softening and melting of coating and the friction ring, with a large number of fragments bonding and being pulled out of the wear interface. The wear process is thus a continuous bonding, peeling and discharging of the wear interface and, therefore, the coefficient of friction is the maximum. The low oxygen content of the surface analyzed according to the EDS (Table 6) results indicates that the worn surface has flaked off before more serious oxidation has occurred. Therefore, three-body wear and abrasive wear were the primary wear mechanisms.

A higher load causes a greater rate of coated surface flaking and wear, as determined by an examination of worn surface morphology and wear processes under various loads. Shifting from adhesive wear and oxidation wear to three-body wear and abrasive wear is how the coating wears.

## 4. Conclusions

To sum up, FeCrSiNiCoC coatings with good performance are produced on 1Cr11Ni heat resistant steel using cutting-edge laser-cladding equipment. In the current study, the effect of temperature and load on the coatings is investigated and the wear mechanism is analyzed. Below is a summary of the principal conclusions drawn from this study:

1. The laser-cladding FeCrSiNiCoC coating is fully dense and homogeneous, without internal defects such as cracks and pores. It has a strong metallic weld with the 1Cr11Ni heat resistant steel substrate. The coating has both dendritic and eutectic structures. The phase constitution is Fe-Cr phase and γ-Fe phase. The average microhardness of the coating is 467 HV_0.5_ ± 22.6 HV_0.5_.
2. FeCrSiNiCoC coating has an exceptional wear resistance at high temperatures. In this study, while the load remains constant at 200 N, the friction coefficient drops as the temperature rises. With increased temperatures, coating wear mechanisms evolved from abrasive wear, adhesive wear and oxidation wear to oxidation wear and three-body wear. At 500 °C, the coating exhibits the best comprehensive wear performance and the lowest wear rate of 2.3 × 10^−5^ g/m.
3. As the load is increased when the temperature is constant at 500 °C, the coating’s wear rate grows progressively, and serious plastic deformation arises on the coating’s surface, although the coating’s friction coefficient alters minimally. The wear mechanism of the coating shifts from adhesive wear and oxidation wear to three-body wear and abrasive wear.

## Figures and Tables

**Table 1:** Nominal chemical composition of FeCrSiNiCoC powder and 1Cr11Ni heat resistant steel substrate.

Name	Mass Fraction/%								
	Cr	Si	Ni	Co	C	Fe	W	Mo	V
FeCrSiNiCoC powder	20	10	4	4	2	Bal.	-	-	-
1Cr11Ni heat resistant steel	11.60	-	1.78	-	0.13	Bal.	1.85	0.47	0.23

**Table 2:** Particle size distribution of different elements.

Powder	D_V_ (10)/μm	D_V_ (50)/μm	D_V_ (90)/μm
Fe	53.5	74.1	104
Cr	17.9	38	63.2
Si	30.9	84.2	138
Ni	53.7	75.1	106
Co	49.5	70.6	101
C	24.1	35.3	56.9

**Table 3:** Wear width, wear depth and wear rate of coatings at various temperatures.

Temperature	Wear Width/(μm)	Wear Depth/(μm)	Wear Rate × 10^−5^/(g/m)
50 °C	6034	98.6	4.3
300 °C	4531	73.5	3.5
500 °C	4159	53.6	2.3
700 °C	7152	152.3	5.4

**Table 4:** Wear width, wear depth and wear rate of coatings at various loads.

Temperature	Wear Width/(μm)	Wear Depth/(μm)	Wear Rate × 10^−5^/(g/m)
100 N	4432	53.4	1.8
200 N	4531	73.5	2.3
300 N	4723	90.6	3.9
400 N	5530	113.4	5.4

**Table 5:** EDS analysis outcomes of worn surfaces of FeCrSiNiCoC coatings at various temperatures.

Temperatures	Atomic Fraction/%						
	Fe	Cr	Si	Ni	Co	C	O
50 °C	64.50	9.86	2.03	0.55	0.66	1.20	21.19
300 °C	60.70	8.32	1.83	0.51	0.66	1.17	26.81
500 °C	62.96	5.87	0.50	0.43	0.47	1.23	28.54
700 °C	67.57	3.42	0.57	0.31	0.35	0.83	26.95

**Table 6:** EDS analysis results of worn surfaces of FeCrSiNiCoC coatings at various loads.

Loads	Atomic Fraction/%						
	Fe	Cr	Si	Ni	Co	C	O
100 N	62.32	4.27	0.24	0.20	0.32	1.67	30.98
200 N	62.96	5.87	0.50	0.43	0.47	1.23	28.54
300 N	62.17	6.15	0.57	0.62	0.67	1.18	28.64
400 N	70.84	6.52	2.72	2.74	0.70	2.36	14.12

## Data Availability

Not applicable.

## Supplementary Materials

The following supporting information can be downloaded at: https://www.mdpi.com/article/10.3390/ma16083263/s1, Figure S1: Surface morphologies of FeCrSiNiCoC coating at different temperatures; Table S1: Chemical composition of FeCrSiNiCoC coatings at different temperatures.
