# Supplementary material for: Effect of Temperature and Load on Tribological Behavior in Laser-Cladded FeCrSiNiCoC Coatings
Source: Materials (Basel). 2023 Apr 21;16(8):3263. doi: 10.3390/ma16083263 (PMC10141079; doi:10.3390/ma16083263)
Supplement: Supplementary file 1 [file materials-16-03263-s001.zip › materials-2264482-supplementary.pdf]

In order to investigate the changes in the organization and structure of the coatings with temperature, the FeCrSiNiCoC coatings were heated to 300 °C, 500 °C and 700 °C in a muffle furnace and maintained for one hour. Figure 3 showed the XRD diffraction pattern of FeCrSiNiCoC coating after different heat treatment temperatures. After heated at 300 °C, the structure of the coating phase changed from γ-Fe, Fe-Cr to α-Fe, Fe-Cr. And when the heat treatment temperature was increased to 500 °C and 700 °C, in addition to α-Fe and Fe-Cr, carbides Cr_7C_3 were produced in the coating.

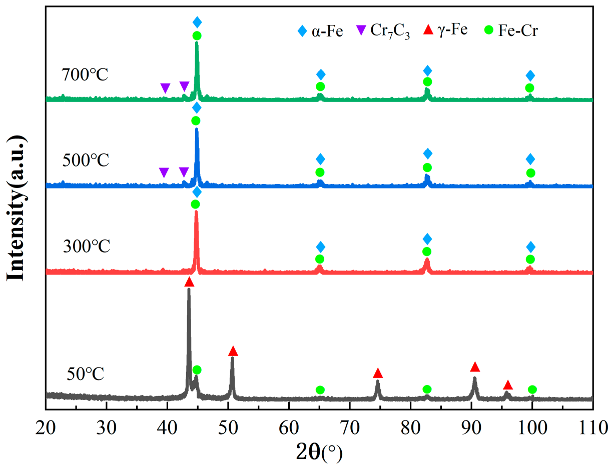

Figure 3. XRD patterns of FeCrSiNiCoC coatings after different heat treatment temperatures

Figure S1 showed the SEM photos of the surface of FeCrSiNiCoC coating after different heat treatment temperatures. Table S1 showed the results of EDS analysis of the surface of FeCrSiNiCoC coatings at different heat treatment temperatures. As the heat treatment temperature was raised, the coating phase structure changed from γ-Fe to α-Fe and the grain size decreased, resulting in an improvement of coating hardness. And the grain size remains unchanged up at 500 °C. According to Figure S1 it can be seen that there were few Cr_7C_3 carbides precipitated at the grain boundaries during the heat treatment temperature of 500 °C, which led to a further increase in wear resistance. This is due to the fact that C is less soluble in α-Fe than in γ-Fe and therefore C precipitates out of the grains making the C content of the grain boundaries rise to produce carbides and increase hardness. When the heat treatment temperature was 700 °C, a large number of carbides were produced at the grain boundaries and the hardness of the coating improved but the brittleness increased, which transformed its wear mechanism into three-body wear and greatly reduced the wear resistance. Therefore, the FeCrSiNiCoC coating had the best wear resistance at 500 °C.

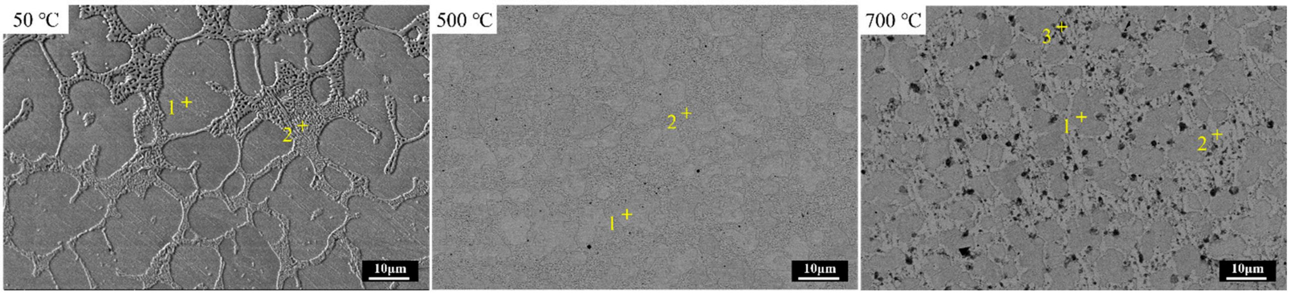

Figure S1. Surface morphologies of FeCrSiNiCoC coating at different temperatures

Table S1 Chemical composition of FeCrSiNiCoC coatings at different temperatures

Temperature	Area	Mass fraction/%						Atomic fraction/%					
		Fe	Cr	Si	Ni	Co	C	Fe	Cr	Si	Ni	Co	C
50 ℃	1	66.47	17.27	6.35	4.94	4.19	0.78	60.38	16.89	11.54	4.28	3.62	3.29
	2	46.72	36.76	6.11	5.00	3.78	1.63	40.81	34.58	10.67	4.17	3.14	6.63
500 ℃	1	75.70	8.59	6.36	4.61	4.24	0.51	69.87	8.51	11.67	4.05	3.71	2.19
	2	63.32	21.56	5.81	4.85	3.59	0.87	57.51	21.03	10.49	4.19	3.09	3.69
700 ℃	1	70.89	13.63	6.53	4.84	3.94	0.17	65.86	13.60	12.07	4.28	3.47	0.72
	2	60.05	24.12	6.59	4.32	3.47	1.45	53.05	22.88	11.57	3.63	2.91	5.96
	3	61.11	22.25	6.09	4.97	3.49	2.08	53.21	20.81	10.55	4.12	2.88	8.43
